# Workplace Discrimination and Burnout Among Asian Nurses in the US

**DOI:** 10.1001/jamanetworkopen.2023.33833

**Published:** 2023-09-14

**Authors:** Jin Jun, Jennifer Kue, Ana Kasumova, Minjin Kim

**Affiliations:** 1 Center for Healthy Aging, Self-Management and Complex Care, College of Nursing, Ohio State University, Columbus; 2 College of Nursing, University of Southern Florida, Tampa; 3 College of Arts and Science, The Ohio State University, Columbus; 4 College of Nursing, University of Cincinnati, Cincinnati, Ohio

## Abstract

This cross-sectional study analyzes the association of workplace discrimination with burnout among Asian nurses in the US.

## Introduction

Racism and discrimination in the workplace predate the COVID-19 pandemic among individuals of different races and ethnicities, including those of Asian ancestry.^1^ The emerging research^2^ highlights the negative effects on clinicians’ well-being from these experiences. Clouded by the model minority myth and the false sense of safety behind professional identity,^3^ there is a critical omission in evaluating Asian nurses’ racial experiences at work.

## Methods

This cross-sectional study followed the STROBE reporting guideline and received exempt status from The Ohio State University institutional review board because the research was in accordance with 45 CFR § 46.104. We obtained participants’ informed consent online at the beginning of the survey, which investigated workplace discrimination and burnout among nurses who self-identified as Asian, including those who descended from original peoples of East Asia, Southeast Asia, the Indian subcontinent, Hawaii, Guam, Samoa, or other Pacific Islands.^4^ We used a self-administered, web-based survey following the AAPOR reporting guideline. We recruited Asian nurses in the US using social media from November to December 2022. Due to the high likelihood of bot-generated illegitimate responses common in social media data, we implemented a 9-step data quality protection protocol (eMethods in Supplement 1).

The primary outcome, burnout, was measured using a validated, single-item questionnaire on a 5-point Likert scale, dichotomized into “definitely/completely burnout” and “no or occasional stress” for analysis.^5^ Perceived workplace discrimination was measured using the Chronic Work Discrimination and Harassment Scale, which included questions about experiencing racial and ethnic slurs, lack of advancement, and unfair treatment in the past 12 months.^6^ The responses were collected on a 5-point Likert scale, which was dichotomized as infrequent vs frequent. Data on individual and work demographics, primary language, self-cultural identity, and nurse-to-nurse teamwork were also collected. Descriptive statistics and logistic regression were conducted using Stata statistical software version 16 (StataCorp) with a 2-sided *P* < .05 denoting statistical significance.

## Results

A total of 236 respondents (mean [SD] age 34.4 [8.4] years; 116 women [49.2%]; 54 Filipino respondents [22.9%]) were included in the analysis. A total of 99 respondents (42.0%) reported definite or complete burnout. Nurses reported that workplace discrimination increased since COVID-19 (before COVID-19, mean [SD] 4.6 [2.8] nurses vs after COVID-19, mean [SD] 5.3 [3.1] nurses). Approximately 75% of respondents (177 respondents [74.2%]) reported experiencing job harassment, unfair treatment, and/or feeling invisible at work a few times a year or more (Table 1).

**Table 1.** Participant Characteristics

Variable	Participants, No. (%)		
	Overall (N = 236)	Men (n = 120)	Women (n = 116)
Individual characteristics			
Age, mean (SD) y	34.4 (8.4)	33.2 (8.31)	35.6 (8.38)
Generation of US residency^a^			
Years in the US, mean (SD)	17.1 (10.7)	15.2 (9.6)	18.6 (11.3)
First	72 (30.5)	32 (27.4)	40 (35.1)
Second	104 (44.1)	57 (48.7)	47 (41.2)
Third or greater	55 (23.3)	28 (23.9)	27 (23.7)
Prefer not to answer	5 (2.1)	3 (2.5)	2 (1.7)
Ethnicity			
Chinese	24 (10.2)	9 (7.5)	15 (12.9)
Filipino	54 (22.9)	27 (23.3)	27 (22.5)
Hawaiian	43 (18.2)	23 (19.2)	15 (12.9)
Indian	10 (4.2)	5 (4.2)	5 (4.3)
Multiple	25 (10.6)	12 (10.0)	13 (11.2)
Other^b^	80 (33.9)	42 (35.0)	35 (30.2)
Primary language spoken with families and friends			
Mostly or only native language	78 (33.2)	48 (40.3)	30 (25.9)
Native language and English equally	77 (32.8)	33 (27.7)	44 (37.9)
Mostly or only English	80 (34.0)	38 (31.9)	42 (36.2)
Self-identity			
Completely Asian or more Asian than American	128 (54.2)	67 (55.8)	61 (52.6)
Asian-American	85 (36.0)	37 (30.8)	48 (41.4)
Completely or more American than Asian	23 (9.8)	16 (13.3)	7 (6.03)
Years in nursing, mean (SD)	8.73 (8.1)	8.5 (8.6)	8.9 (7.4)
Years in current position, mean (SD)	4.72 (4.9)	4.9 (4.8)	4.6 (4.9)
Nursing degree			
Associates degree or diploma	38 (16.1)	22 (18.5)	16 (13.8)
Bachelor of science in nursing	88 (37.2)	38 (31.9)	50 (43.1)
Masters or doctorate	109 (46.2)	59 (49.6)	50 (43.1)
Work characteristics			
Employer type			
Hospital	81 (34.3)	42 (35.0)	39 (33.6)
Critical care	24 (29.6)	13 (31.0)	11 (28.2)
Step down unit, medical, or surgical	30 (37.0)	20 (47.6)	10 (25.6)
Emergency department	13 (16.1)	3 (7.1)	10 (25.6)
Other	14 (17.3)	6 (14.3)	8 (20.5)
Long-term care, home health, or specialty care	52 (22.0)	24 (20.0)	28 (24.1)
Public health or school nursing	69 (29.2)	31 (26.7)	38 (31.7)
Outpatient ambulatory care	21 (8.9)	12 (10.3)	9 (7.5)
Other (eg, military)	13 (5.5)	6 (5.2)	7 (5.8)
Role			
Staff nurse	143 (60.6)	75 (62.5)	68 (58.6)
Nurse manager or director	28 (11.9)	18 (15.0)	10 (8.6)
Advanced practice nurse (eg, nurse practitioner)	23 (9.8)	13 (10.8)	10 (8.6)
Case manager or clinical coordinator	33 (14.0)	13 (10.8)	20 (17.2)
Other	9 (3.8)	1 (0.8)	8 (6.9)
Job status			
Regular or permanent full time (>36 h/wk)	171 (72.5)	84 (70.0)	87 (75.0)
Regular or permanent part time (≤36 h/wk)	56 (23.7)	33 (27.5)	23 (19.8)
Traveler contract	9 (3.8)	3 (2.5)	6 (5.2)
No. of hours worked in the last 7 d, mean (SD)	37.5 (12.7)	37.6 (11.9)	37.5 (13.5)
Intent to leave position			
No intent to leave	133 (56.4)	62 (53.5)	71 (59.2)
Within the next 6-12 mo	22 (9.3)	13 (11.2)	9 (7.5)
Undecided	81 (34.3)	41 (35.3)	40 (33.3)
Intent to leave profession			
No intent to leave	177 (75.0)	85 (73.3)	92 (76.7)
Within the next 6-12 mo	8 (3.4)	4 (3.5)	4 (3.3)
Undecided	51 (21.6)	27 (23.3)	24 (20.0)
Nurse-to-nurse interaction			
Great deal of teamwork (agree)	183 (77.7)	94(78.3)	89(76.7)
Supportive to each other (agree)	196 (83.1)	99 (82.5)	97 (83.6)
Pitch in and help each other (agree)	177 (75.0)	89 (74.2)	88 (75.9)
Burnout			
No burnout but occasional stress	137 (58.1)	71 (59.2)	66 (56.9)
Definite or complete burnout	99 (42.0)	49 (40.8)	50 (43.1)
Resilience score, mean (SD)^c^	28.1 (5.8)	28.9 (5.8)	27.3 (5.9)
Experienced discrimination at work, mean (SD)			
Before COVID-19	4.6 (2.8)	4.9 (2.8)	4.3 (2.7)
After COVID-19	5.3 (3.1)	5.5 (3.2)	5.2 (3.1)
Type and frequency of work discrimination^d^			
Job harassment^e^			
Infrequent	83 (35.2)	36 (30.0)	84 (70.0)
Frequent	153 (64.8)	47 (40.5)	69 (59.5)
Unfair treatment at work^f^			
Infrequent	80 (33.9)	38 (31.7)	82 (68.3)
Frequent	156 (66.1)	42 (36.2)	74 (63.8)
Feeling invisible at work^g^			
Infrequent	102 (43.2)	51 (42.5)	69 (57.5)
Frequent	134 (56.8)	51 (43.0)	65 (56.0)

^a^ First generation refers to those born outside of the US; second generation refers to the US-born children of foreign-born parents; and third generation refers to US-born children of US-born parents.

^b^ Other includes Bangladeshi, Bhutanese, Bruneian, Burmese, Cambodian, Carolinian, Chamorro, Chuukese, Guamanian, Hmong, Japanese, Korean, Laotian, Malaysian, Maldivian, Nepali, Pakistani, Papua New Guinean, Samoan, Singaporean, Sri Lankan, Thai, Taiwanese, and Vietnamese.

^c^ Resilience measured using the 10-item Connor-Davidson Resilience Scale with scores ranging from 0 (least resilient) to 40 (most resilient).

^d^ All items were measured on a 5-item Likert scale ranging from never to at least once a week; infrequent was defined as never or less than once a year and frequent was defined as more than a few times a year.

^e^ Items for job harassment measured the frequency of racial or ethnic slurs used by supervisors or coworkers.

^f^ Items for unfair treatment at work measured the frequency of being watched closely, being humiliated at work, and being treated unfairly compared with others.

^g^ Items for feeling invisible at work measured the frequency of being ignored, being assumed to be a lower status, or being denied promotion.

In multivariable analysis (Table 2), those who reported frequent discrimination at work were more likely to have burnout compared with those who experienced infrequent discrimination (adjusted odds ratio [aOR], 2.67; 95% CI, 1.31-4.88; *P* = .001). Respondents who primarily spoke English were also more likely to report burnout compared with those who primarily spoke their native language (aOR, 2.44; 95% CI, 1.10-5.45; *P* = .04). Working more than 40 hours a week was also associated with increased likelihood of burnout (aOR, 2.10; 95% CI, 1.09-4.09; *P* = .01).

**Table 2.** Association of Work Discrimination With Burnout of Asian Nurses in the US

Variable	Adjusted odds ratio (95% CI)
Sex	
Male	0.79 (0.43-1.45)
Female	1 [Reference]
Age	0.98 (0.94-1.02)
Generation of US Residency^a^	
First	1 [Reference]
Second	0.78 (.36-1.69)
Third or more	0.59 (.24-1.44)
Primary language spoken with families and friends	
Mostly or only native language	1 [Reference]
Native language and English equally	1.78 (0.81-3.89)
Mostly or only English	2.44 (1.10-5.45)^b^
Self-cultural identity	
Completely or more Asian than American	1 [Reference]
Asian-American	1.53 (0.79-2.96)
Completely or more American than Asian	1.43 (0.48-4.26)
Nursing degree	
Associate degree or diploma	1 [Reference]
Bachelor of science in nursing	0.44 (0.18-1.08)
Masters or doctorate	0.55 (0.23-1.36)
Role	
Staff registered nurse	
Non-staff registered nurse	0.71 (0.37-1.36)
Facility	
Hospital	1 [Reference]
Outpatient or ambulatory	0.99 (0.46-2.10)
Long-term care	0.69 (0.33-1.41)
No. of hours worked in the last 7 d	
≤40	1 [Reference]
>40	2.10 (1.09-4.09)^b^
Job status	
Full time permanent	1 [Reference]
Part-time permanent	0.88 (0.42-1.87)
Contract, traveler, or agency	0.14 (0.01-1.41)
Teamwork between nurses	
Disagree	1 [Reference]
Agree	1.65 (0.77-3.62)
Work harassment^c^	
Infrequent	1 [Reference]
Frequent	2.67 (1.31-4.88)^d^

^a^ First generation refers to those born outside of the US; second generation refers to the US-born children of foreign-born parents; and third generation refers to US-born children of US-born parents.

^b^ *P* < .05.

^c^ Infrequent was defined as never or less than once a year and frequent was defined as more than a few times a year.

^d^ *P* < .01.

## Discussion

Our findings in this cross-sectional study contribute to the growing empirical evidence indicating that racial discrimination plays a substantial role in burnout among Asian nurses. Previous studies^2^ on burnout showed lower burnout rates among Asian clinicians. Although we could not ascertain these differences by race, we found English as the primary language was associated with burnout, suggesting potential cultural or linguistic associations with burnout measures.

The small sample size, self-selective nature of convenience sampling, high proportion of participants with advanced degrees, and overrepresentation of men in the sample limited the generalizability of our findings. Workplace discrimination is associated with a detrimental decrease in the well-being of Asian nurses, highlighting the issue of anti-Asian racism. Building a knowledge base presents an opportunity as an initial step toward integrating and engaging Asian nurses in antiracism efforts within the workplace.

## Notes

Supplement 1. **eMethods.** Steps for Detecting Bots, Fraudulent, or Suspicious Responses From Social Media Data Collection

Supplement 2.
Data Sharing Statement
