# Supplementary material for: Workplace Discrimination and Burnout Among Asian Nurses in the US
Source: JAMA Netw Open. 2023 Sep 14;6(9):e2333833. doi: 10.1001/jamanetworkopen.2023.33833 (PMC10502516; doi:10.1001/jamanetworkopen.2023.33833)
Supplement: Supplement 1. — eMethods. Steps for Detecting Bots, Fraudulent, or Suspicious Responses From Social Media Data Collection [file jamanetwopen-e2333833-s001.pdf]

Supplemental Online Content

Jun J, Kue J, Kasumova A, Kim M. Workplace discrimination and burnout among Asian nurses in the US. *JAMA Netw Open.* 2023;6(9):e2333833. doi:10.1001/jamanetworkopen.2023.33833

eMethods. Steps for Detecting Bots, Fraudulent, or Suspicious Responses From Social Media Data Collection

This supplemental material has been provided by the authors to give readers additional information about their work.

© 2023 Jun J et al. *JAMA Network Open.*

eMethods. Steps for Detecting Bots, Fraudulent, or Suspicious Responses From Social Media Data Collection

There are two main types of concerning responses when collecting data using social media. Bot or fraudulent responses are responses by Bot or web, which are an artificial intelligent response that intent to imitate human activity on the internet. Another type of fraudulent responses are suspicious types in which person(s) provide duplicate or false responses using similar emails for incentives. We implemented a multi-pronged approach in the survey development and data management to create a strong bot detection and protection process to minimize the number of potential bot or suspicious responses and to allow us to easily identify fraudulent responses.

A. Data Protection Setting Prior to Data Collection

We utilized the internal survey protection settings available on Qualtircs as listed below to prevent ballot box stuffing (a tool that places a cookie in the browser once a person has submitted a response).

1. reCAPTCHA (Completely Automated Public Turing Test to tell Computers and Humans Apart) scores (a question placed prior to the survey asking the respondent to identify certain items in pictures or replicate a series of letters),
    - Applied Filter, “reCAPTCHA if greater than 0.5
2. Bot detection bot detection (a Qualtrics survey question that indicates a reCAPTCHA score that relates to the probability that the respondent is a bot
3. HTTP referrer verification HTTP referrer verification (an option that verifies all responses come from a specific link) were activated at the launch of the survey, sophisticated bots were able to bypass these protective measures.

B. Data Cleaning Steps for Bot and Fraudulent Detection

4. Abnormal duration to complete the surveys
    - We removed outlier response times, defined as under 5 minutes (300 seconds) or 30 minutes (1,800 seconds). We selected this time limits based on the average time during the pilot testing of the survey, which was 12 minutes and 27 seconds.
5. Bots determined by Qualtrics
    - Removed responses with a reCAPTCHA score less than 0.5 from Google’s reCAPTCHA V3 as suggested by the Google’s developer guide (Google Developers 2020; Qualtrics 2021)
6. Fraud determined by Qualtrics
    - Removed RelevantIDFraudScore (range 0-130, Qualtrics recommends 30), we removed responses if RelevantIDFraudScore was greater than or equal to 75.
    - FraudID was not turned on until the second day of data collection. The data collected prior to October 2nd did not have FraudID. If no RelevantIDFraudScore was captured, we kept the responses and manually reviewed the responses.
7. Duplicates determined by Qualtrics
    - Removed RelevantIDDupplicate Score greater than 75.
8. Suspicious detection
    - Removed non-US countries using latitude and longitude codes captured in Qualtric.
    - Screened open text qualitatively for repeated, nonsense, and patterned responses
    - Screened for conflicting data

© 2023 Jun J et al. *JAMA Network Open.*

- ✓ The number of hours worked in the previous week greater than 40
- ✓ Duration as a nurse vs. duration in the current job
- ✓ Age vs. duration as a nurse
- ✓ Rare ethnicity

- Screened for similar or same emails

9. When the above steps fail and the research cannot determine or agree on the origin of responses, we contacted participants using the email provided to confirm that they were not bot.

© 2023 Jun J et al. *JAMA Network Open.*
